# A clinically validated whole genome pipeline for structural variant detection and analysis

**DOI:** 10.1186/s12864-019-5866-z

**Published:** 2019-07-16

**Authors:** Nir Neerman, Gregory Faust, Naomi Meeks, Shira Modai, Limor Kalfon, Tzipora Falik-Zaccai, Alexander Kaplun

**Affiliations:** 1 Variantyx Inc, Framingham, MA 01867 USA; 2 Institute of Human Genetics, Galilee Medical Center, Naharia, Israel; 3 The Azrieli Faculty of Medicine, Bar Ilan, Safed, Israel

**Keywords:** Whole genome sequencing, Structural variants, Clinical validation, Pipeline, Diagnostic console, WGS, CNV, Deletion, Duplication, Break point

## Abstract

**Background:**

With the continuing decrease in cost of whole genome sequencing (WGS), we have already approached the point of inflection where WGS testing has become economically feasible, facilitating broader access to the benefits that are helping to define WGS as the new diagnostic standard. WGS provides unique opportunities for detection of structural variants; however, such analyses, despite being recognized by the research community, have not previously made their way into routine clinical practice.

**Results:**

We have developed a clinically validated pipeline for highly specific and sensitive detection of structural variants basing on 30X PCR-free WGS. Using a combination of breakpoint analysis of split and discordant reads, and read depth analysis, the pipeline identifies structural variants down to single base pair resolution. False positives are minimized using calculations for loss of heterozygosity and bi-modal heterozygous variant allele frequencies to enhance heterozygous deletion and duplication detection respectively. Compound and potential compound combinations of structural variants and small sequence changes are automatically detected. To facilitate clinical interpretation, identified variants are annotated with phenotype information derived from HGMD Professional and population allele frequencies derived from public and Variantyx allele frequency databases. Single base pair resolution enables easy visual inspection of potentially causal variants using the IGV genome browser as well as easy biochemical validation via PCR. Analytical and clinical sensitivity and specificity of the pipeline has been validated using analysis of Genome in a Bottle reference genomes and known positive samples confirmed by orthogonal sequencing technologies.

**Conclusion:**

Consistent read depth of PCR-free WGS enables reliable detection of structural variants of any size. Annotation both on gene and variant level allows clinicians to match reported patient phenotype with detected variants and confidently report causative finding in all clinical cases used for validation.

**Electronic supplementary material:**

The online version of this article (10.1186/s12864-019-5866-z) contains supplementary material, which is available to authorized users.

## Background

Short read based Whole Genome Sequencing (WGS) is slowly but surely becoming an integral part of the landscape of clinical diagnostic testing for rare genetic disorders. However, in current clinical practice WGS is mainly still used as ‘enhanced’ Whole Exome (WES). Indeed, due to its uniformity and lack of pull down or amplification artifacts, WGS typically provides better than WES coverage of coding and adjacent regulatory regions. This approach, however, ignores many of the advantages of WGS which provides unique opportunities for detection of structural variants (SVs), pathologic short tandem repeats and mitochondrial variants, which otherwise require separate assays. In particular, disease causing SVs in medical genetics are detected by karyotyping [1] and chromosomal microarrays (CMAs) [2]. However, these methods are limited in resolution and cannot identify all types of SVs.

SVs are a diverse group of variants which consists of copy number variants (CNVs), namely duplications or deletions of human genetic sequences resulting in an abnormal number of alleles; insertions of foreign genetic sequences, such as transposons; balanced translocations and inversions. A typical genome includes many thousands of such genetic aberrations [3, 4], and it is challenging not only to identify them but also to determine which, if any, are causative to the patient’s phenotype.

While detection of small sequence changes has become fairly standardized using “gold standard” tools such as BWA [5] and GATK [6] which are almost universally used for sequence alignment and variant calling, the situation for SV detection is quite different. There are multiple tools and pipelines designed for detection and reporting of SVs basing on short read WGS data ([7–10] among others), however there has been no coalescence around a single consensus calling pipeline, and none of them have been utilized in clinical diagnostic testing.

Here we report a structural variant component of a comprehensive WGS-based clinical test for diagnostics of rare genetic disorders caused by germline genetic variants, developed by Variantyx. The test as a whole, including the structural variant part, underwent analytical and clinical validation, College of American Pathologists accreditation and proficiency testing, and is certified by CLIA. The SV component of Variantyx Genomic Intelligence pipeline uses a combination of breakpoint analysis (using split and discordant reads) and read depth analysis to identify structural variants, often down to single base pair resolution. False positives are minimized using ancillary calculations such as loss of heterozygosity and bi-modal heterozygous variant allele frequencies. Identified variants are annotated with phenotype information derived from HGMD Professional and population allele frequencies derived from DGV and Variantyx PAF database facilitating clinical interpretation. Single base pair resolution enables easy visual inspection of potentially causal variants using the IGV genome browser. U.S. board certified clinical geneticists use online Diagnostic Console to reviews results, select appropriate variants and generate clinical report.

## Results and discussion

**Fig. 1:**
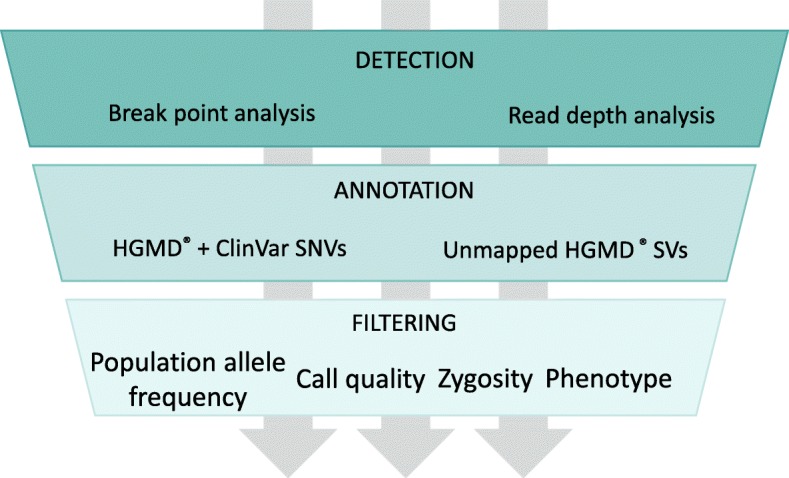
SV component of Variantyx Genomic Intelligence WGS analysis pipeline

The SV component of Variantyx Genomic Intelligence Whole Genome Sequencing analysis workflow is comprised of three major parts: variant detection, annotation and filtering (Fig. 1).

### Variant detection

The SV detection pipeline is generally organized as has been previously published [11, 12]. While some of the tools are used as published, others are significantly redesigned and improved, and still others entirely new. In addition, the results of the raw variant calls have been augmented and filtered using in-house developed annotations, techniques, and data sources.

In general, structural variants can be divided into two categories: those resulting in unbalanced changes in number of copies of human DNA, and those resulting in balanced changes so the total number of copies remains the same. Copy number variants (CNVs), which include deletions, duplications and unbalanced translocations of different sizes, can be detected by two approaches: depth-based analysis and break point analysis [13]. The first is to identify regions in which read depth is significantly different from typical depth in same region in samples which are known not to have copy number variation in this region. The other is to look at variant edges and detect split reads (where two portions of a single read map to two distinct locations in the reference) and discordant reads (where paired reads map in positions or direction inconsistent with expected basing on insert size used). Only break point analysis can be used to identify balanced SVs, including inversions, translocations, as well as insertions of foreign DNA such as transposable elements.

Both read depth and break point signals are utilized by Variantyx Genomic Intelligence algorithms, while results on larger break point derived variants must be confirmed by depth signal to be considered a true positive. Structural variants are called with the use of Samblaster [11] for read extraction, LUMPY [14] for read-based SV calling and SVtyper [11] for genotyping, using default parameters. These calls are then combined with Variantyx depth caller CNVs to form a union of calls. The depth calling algorithm utilizes a proprietary model built with known true negative WGS samples sequenced and aligned under the same conditions. The rolling average read depth-based model rolls up 100 bp segments into buckets of 10,000 and 2500 bp. We found these sizes optimal while 10,000 bp bucket allows to detect uninterrupted stretches of read depth deviation in larger CNVs and 2500 bp bucket allows to detect smaller CNVs and improve exact position of larger ones. Break point analysis allows detection of smaller SVs and all types of balanced variants.

The most common SVs, deletions and tandem duplications, have a single break point which exhibits in the sample reads the unexpected juxtaposition of two noncontiguous reference coordinates marking the start and end of the structural event. These have a readily identified signature and are easy to classify. Other events such as insertion of DNA naturally have two break points, one at the start and the end of the inserted fragment. However, in such cases, one of the breakpoints may not be detected, because the number of split or discordant reads supporting the second breakpoint does not reach calling threshold, or the second breakpoint is located in a difficult to map region containing, for example, highly repeated sequences. This is especially true if the inserted element is a transposon. Translocation of chromosome arms also have one break point but are hard to distinguish from an insertion with an undetected second break point. Thus, even an unclassified single breakpoint could indicate a potentially disruptive SV and such variants are annotated and subsequently uploaded to the Diagnostic Console together with the pre-classified SVs.

While exact quantitate benchmarking of the variant calling results by Variantyx pipeline relative to other SV calling tools and algorithms has not been performed, some comparison could be made. Most available tools use either read-based or depth-based calling, while our approach is to merge calls from both read-based and depth-based callers to increase sensitivity. For example, SOAPsv [15] and LUMPY are breakpoint detection based. We use machine learning algorithm to detect CNVs based on large number of human genomes sequenced under same standard operating procedures, resulting in highly repeatable normalized read depth. This approach provides significantly better results than CNVnator or Control-FREEC [16] which run only one sample at a time and have no prior knowledge of expected coverages.

Raw output of the SV calling pipeline includes significant number of false positives that must be removed prior to introduction to the Diagnostic Console. Many of these false positive calls can be filtered out based on a number of criteria specific to variant type. In particular, all variants called based on break point analysis must be supported by at least 20 observations (combined split and discordant reads), out of which 5 must be split reads. In addition, CNVs over 5000 bp long called using break point analysis must have at least 30% overlap with those called using read depth analysis.

**Fig. 2:**
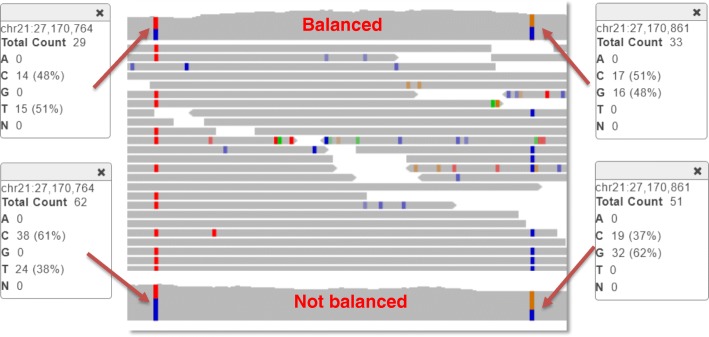
Ratio of balanced (between 40 and 60%) out of total overlapping SNVs is shifted in case of heterozygous duplications

For further removal of false positive calls, we examine the detected Single Nucleotide Variants (SNVs) within the SV region, and apply the following thresholds for three types of SVs:

1. Homozygous deletion must overlap no more than 1 SNV per 1000 bp length, with minimum of 5 SNVs to apply the rule. This rule is based on the fact that in most regions of the genome (with notable exceptions such as sex chromosomes) the frequency of SNVs is higher, and if at least one allele is present the threshold will be exceeded. Ideally, true Alternative deletion should have no SNVs called within its range. Unfortunately, typically it is not the case (particularly in large deletions) since some of the reads are still getting aligned within such deletion, and some SNVs could be called. Thus, Alternative deletions are not filtered out if they intersect some SNVs with frequency no exceeding 1/1000 bp.
2. The fraction of heterozygous SNVs overlapping heterozygous deletion must not exceed 20% of total SNVs called. When two alleles of DNA are present, typically number of heterozygous SNVs significantly exceeds number of alternative SNVs. According to gnomAD data, globally het/alt SNVs ratio is 1.6. Thus, this parameter provides very reliable indication that there is loss of heterozygosity and the heterozygous deletion is indeed present. In typical true Heterozygous deletions long enough to include statistically significant number of SNVs the observed ratio does not exceed 10%.
3. Balanced heterozygous SNVs overlapping duplications must represent no more than 20% of total number of duplications. Balanced heterozygous SNV is defined as one having fraction of reads supporting each of the two alleles between 40 and 60% (See Fig. 2). Since natural 50% balance between alleles is shifted in case of duplication this parameter represents yet another reliable threshold.

Typically, 6 to 8 thousand SVs are called by Variantyx pipeline per genome, while approximately 70% of these SVs pass the default filtration settings. We have analyzed calling and filtration results using the best available to date true SV data set based on Genome in a Bottle sample NA24385 (See Tables 1, 2 and 3). The application of filtration allows to remove most of false positive calls, while leads to loss of relatively small number of true positive variants (removes 38 TP, 297 FP in the analyzed buckets). The most significant impact is on largest bucket, removing all 84 false positive and keeping all 5 true positive SVs. We have also analyzed the effect of individual filters (data not shown). The most impactful filter was the fraction of heterozygous SNVs overlapping heterozygous deletion. Its application removed 47 false positives from the 100,000+ bucket and 40 false positives from the 10,000–100,000 bucket. This filter also removed 4 true positive variants from the 10,000–100,000 bucket. It is important to note that truth data set has its limitations and some “true positives” and “true negatives” are not necessarily such.

**Table 1:** ^a^Variant calling results: before filtering

bucket	TP	FP	FN	sensitivity
100–1000	2233	202	965	0.69825
1000-10,000	613	139	61	0.90950
10,000–100,000	48	97	3	0.94118
100,000+	5	84	0	1

^a^ The analysis has been performed using Genome in a Bottle truth dataset NA24385 (http://tinyurl.com/GIABSV06). Since the truth set was built on top of hg19, we had to lift over results from hg19 to hg38 which dropped some variants. In addition, in order to not be biased we have only compared SVs that were liftable from hg38 to hg19 (to make sure we don’t have any false, false positives). We also removed calls made in non-unique areas and calls < 100 bp

**Table 2:** Variant calling results: after default filtering

bucket	TP	FP	FN	sensitivity
100–1000	2209	142	989	0.69074
1000-10,000	603	60	71	0.89466
10,000–100,000	45	23	6	0.88235
100,000+	5	0	0	1

**Table 3:** Variant calling results: Breakpoint based (LUMPY) only: after default filtering

bucket	TP	FP	FN	sensitivity
100–1000	2209	142	989	0.69074
1000-10,000	570	16	104	0.84570
10,000–100,000	35	5	15	0.7
100,000+	4	0	1	0.8

### Annotation

All the SVs are annotated with information on the variant and gene level. Variant level annotation is comprised of population frequency and pathogenicity data. PAF data is derived from DGV [17] and from Variantyx internal database. HGMD Professional [18] is used for annotation with overlapping pathogenic SVs. HGMD Professional database includes records of over 220,000 pathogenic genetic variants collected by manual curation of peer-reviewed literature. It is very well known and represents industry standard in clinical genetics of small sequence changes, however despite the fact it includes over 20,000 curated pathogenic SV currently it is not widely used in SV annotation. The reason for that is lack of genomic coordinates of the SVs included in HGMD Professional, making the data not readily available for annotation. We have revisited all SV records in HGMD Professional and have supplemented them with genomic coordinates where possible, which allowed utilization of this data in annotation. It the process of annotation SVs overlapping over 70% of known pathologic SVs are considered having variant level pathogenicity annotation. Same strategy is applied for pathogenic SVs reported in ClinVar [19].

**Fig. 3:**
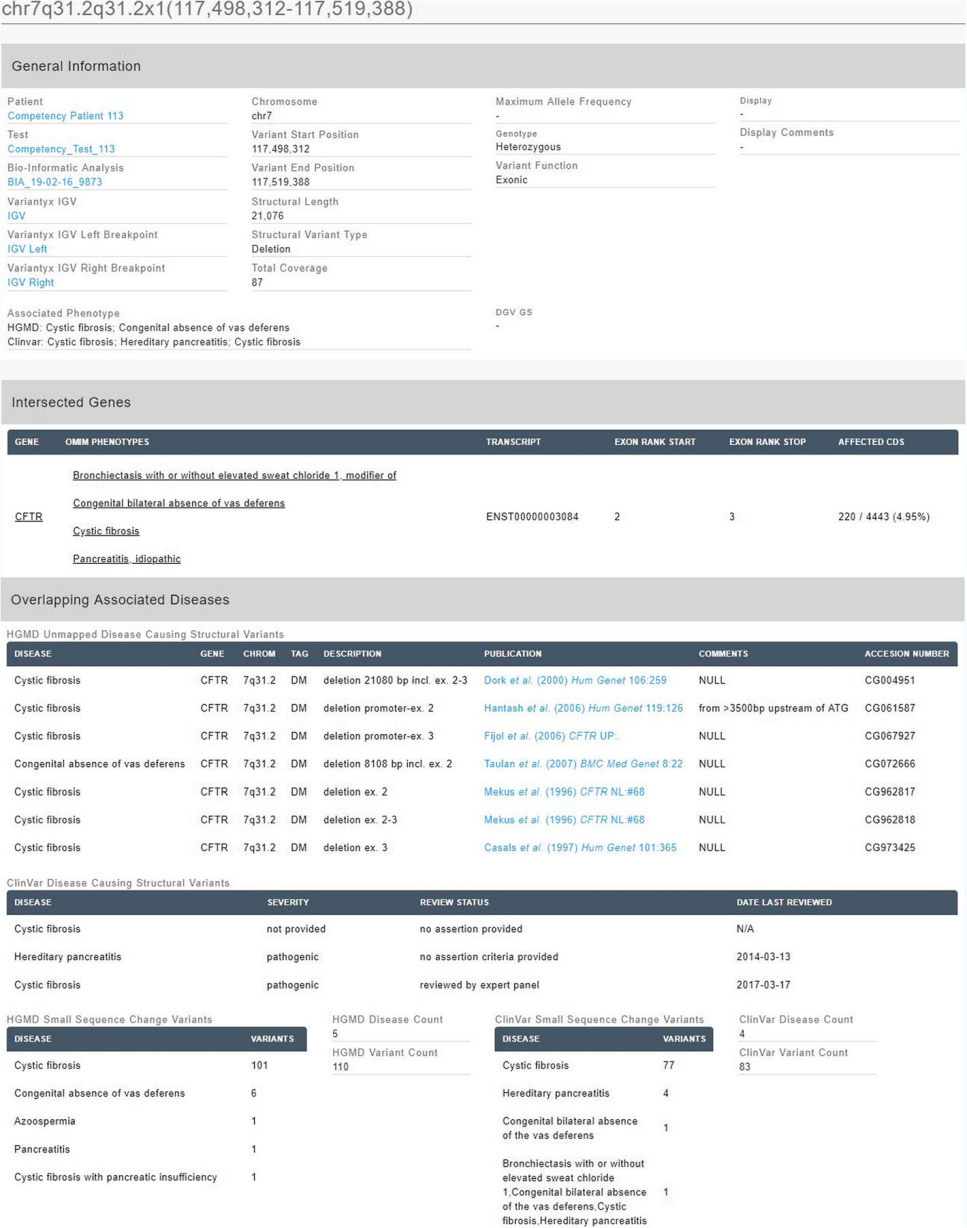
Structural variant annotation on variant and gene level as presented in Diagnostic Console

It often happens that no SVs similar to one detected have been previously reported in peer-reviewed literature, however the SV intersects gene(s) or region with known pathogenic small sequence changes. Data on such changes is derived from HGMD Professional and from ClinVar and complemented with information on known pathogenic genes from OMIM [20] and Orphanet [21]. Annotation with this data is considered gene level and is noted as such. Gene level annotation play an important role in SV pathogenicity classification, particularly in cases of unclassified SVs. Example of pathologic SV with variant and gene level annotation as seen in Variantyx Diagnostic Console is shown in Fig. 3.

Recessive structural variants can be compound to small sequence changes, and detection of such combinatory compound heterozygous pairs is often challenging. To facilitate this process, we have included information on existing complementary SV in annotation of small sequence variants. Such compound (in case of family analysis when paternal and maternal alleles are identifiable) and potential compound (when one of the variants is de novo or patient is tested as a singleton) pairs are presented in a dedicated section of Diagnostic Console, along with compound pairs of small sequence variants.

### Filtration

**Fig. 4:**
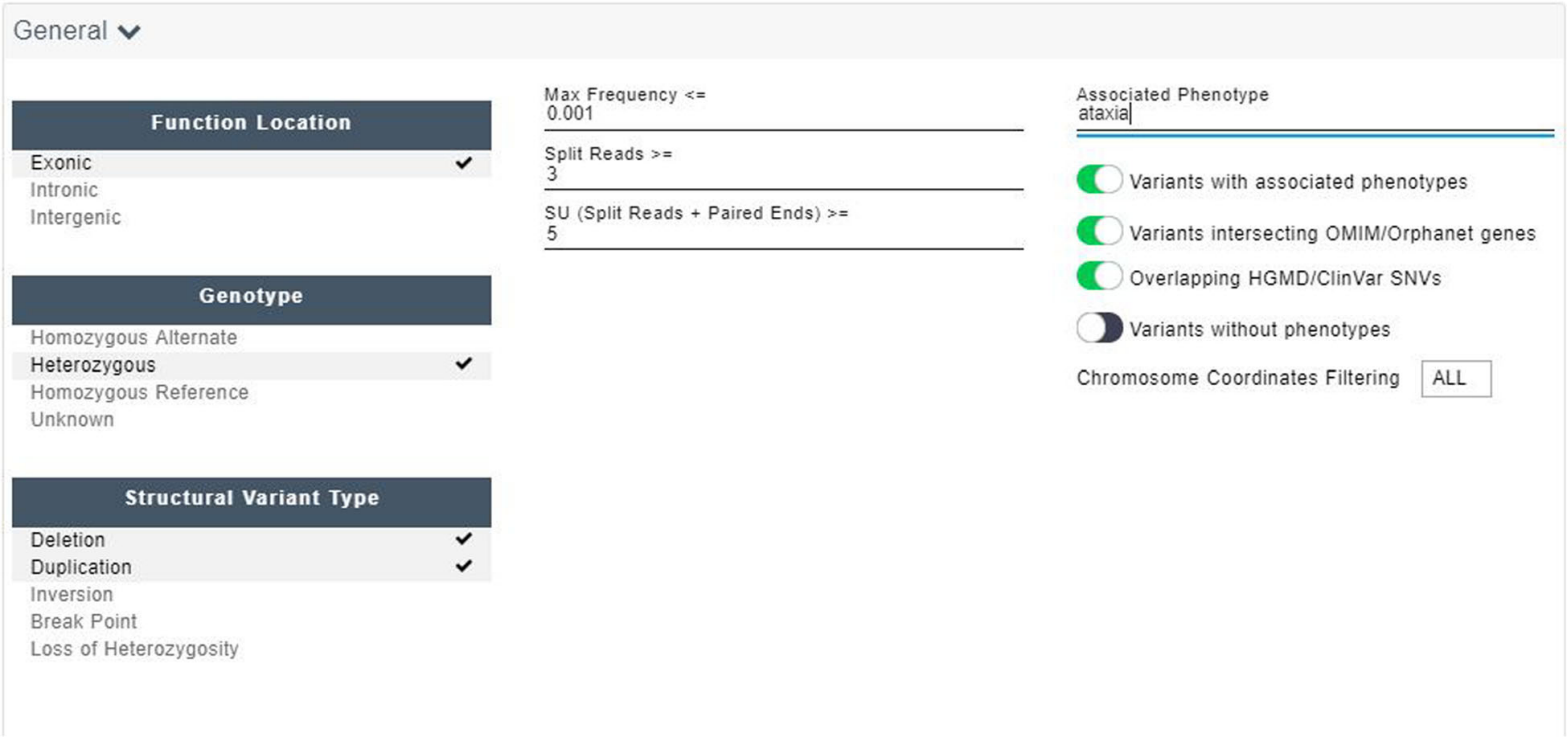
Structural variant filtration in Diagnostic Console

The annotated SVs are uploaded into Diagnostic Console where they can be filtered by the interpreting geneticist. Many parameters are available, but the most important and frequently used for the diagnostic process are variant and gene level phenotype association, population frequency and function location (see Fig. 4). Default parameters are below 2% population frequency (maximum between DGV and Variantyx internal database) and only include variants with associated phenotype on variant level. On second stage variants intersecting OMIM/Orphanet genes and Overlapping HGMD/ClinVar SNVs are analyzed. No changes in technical parameters such as number of split reads or depth call overlap are recommended as part of Unity test structural variants Diagnostic Process (Additional file 1: Method S1).

### Validation

Typically, clinical genetic assay validation would include two phases, analytical and clinical validation. Analytical validation includes comparison of variants called by the assay with known true positive set of variants to determine sensitivity, specificity and positive predicted value. In NGS based genetic test development the industry standard is to use Genome in a Bottle samples, such as GM12878 [22, 23]. Indeed, in case of small sequence changes available true positive variant sets can be used for accurate benchmarking. See Additional file 1: Figure S2 for analytical validation statistics of small sequence changes component of Variantyx Unity test.

Unfortunately, no true positive variant set of acceptable quality is available for analytical validation of SVs. Different sequencing and variant calling methods applied by different research groups produce sets of “true positive” variants which vary between each other by nearly an order of magnitude in terms of quantity and overlap of detected variants [23, 24]. Close examination of a representative group of “true positive” SVs called by different approaches revealed large number of false positives and false negatives, making use of this data unacceptable in analytical validation of clinical test. Thus, we have decided to directly pursue clinical validation of Unity test. To perform clinical validation, we have gathered a statistically significant number of true positive (those having causative pathogenic SV confirmed by orthogonal detection techniques) and true negative clinical samples (those of healthy individuals or affected but having causative genetic variants of different than SV types). Majority of the true positive samples were obtained from public collections, while some originated from different sources [25].

A total of 60 clinical validation cases underwent complete Unity test cycle, starting from *de novo* WGS sequencing all the way to clinical interpretation by board certified clinical geneticists and generation of patient report. No identifying details were disclosed beside patients’ phenotypes, and for healthy controls realistic phenotypes and anamneses were added. In addition to these 60 patient samples, a synthetic sample with a variety of hard to detect pathogenic variants (including two SVs) has been analyzed. Due to large number of detected pathologic genetic variants it was impossible to pass the synthetic sample for real patient data; thus, it is not included in total statistics despite the fact that both pathologic SVs were successfully identified and reported as such. Additionally, three trisomy samples were included. However, since ploidy analysis is performed by Variantyx Genomic Intelligence pipeline on early stage of data analysis and data of patients positive for ploidy aberrations are not uploaded to Diagnostic Console, these samples are also not included in the total while all three were successfully identified by our system.

Out of 60 clinical validation patients, 17 were true positive for pathogenic SV, in some cases with multiple SVs present, and in others the SV was a compound heterozygote with recessive small sequence changes. All but one SV were successfully identified and reported as such. The one missed has been identified by the Diagnostic Console but was not included in the report due to detection of two known pathogenic SNVs that could explain patient phenotype without involvement of SV. In general, significant number of true positive samples found in public repositories belong to cases diagnosed nearly two decades ago by rather narrow, by today’s standards, techniques and might thus include significant pathogenic variants overlooked by original submitters.

Between all patient and synthetic samples which underwent clinical interpretation there were a total of 25 SVs, all of which were detected by Variantyx Genomic Intelligence platform and 24 were clinically reported, resulting in 96% clinical sensitivity for detection of pathogenic SV. It is important to note that true positive samples that include SVs beyond the scope of Variantyx Unity test, such as balanced translocations, were not included in clinical validation.

## Conclusions

The uniformity and consistent read depth of PCR free WGS allows reliable detection of SVs and clinical utilization of SV workflow as part of comprehensive WGS based genetic testing that could be used as the first line diagnostic test. Unity test developed by Variantyx, CLIA certified and CAP accredited for High Complexity Testing, has been clinically validated to serve as clinical use medical genetics assay. The test successively detects SV variants of multiple types, with examples of reported pathologic variants range from 45 bp deletion (see Additional file 1: Figure S1) to complex rearrangement involving millions base pairs on three different chromosomes [26]. While some types of SVs, such as balanced translocations occurring in non-uniquely mappable areas, are still represent a challenge for short read-based test, better resolution and absence of variant size limitation, together with declining sequencing costs, allow WGS based test to be viable alternative to traditional array-based assays.

## Methods

Patient blood or saliva is collected using Variantyx Unity collection kits. DNA is purified and NGS library is prepared using Illumina PCR-Free Truseq nano DNA WGS (550 bp insert size protocol) kit according to manufacturer’s instructions. Sequencing is performed using CLIA approved protocols using Illumina HiSeq X and Novaseq Sequencing machines. FASTQ files are downloaded and processed with Variantyx Genomic Intelligence pipeline. Only tests passing quality threshold parameters for data integrity, contamination, mapping quality etc., (see Additional file 1: Table S2 for complete list of threshold parameters) undergo bioinformatic analysis and clinical interpretation. The interpretation is performed by US board certified clinical geneticists according to clinical diagnostics protocol approved by CAP (see Additional file 1: Method S1 for SV portion of the protocol). Causative variants fitting reporting criteria are included in clinical report, which is submitted to the ordering clinician. A synthetic DNA sample for Unity test validation was purchased from SeraCare (Seraseq Inherited Cancer DNA Mix v1).

## Additional files

Additional file 1: **Table S1** Samples for clinical validation of Variantyx Unity test. **Table S2** Variantyx Unity test thresholds. **Figure S1** Causative heterozygous deletion of 45 bp detected and reported by Variantyx Unity test. **Figure S2** Analytical validation statistics of small sequence changes by Variantyx Unity test basing on combination of 3 different Genome in a Bottle samples. **Method S1** Variantyx diagnostic procedure for reporting pathogenic structural variants. (DOCX 152 kb)

## Data Availability

All the data supporting the results of this article are included within the article. Complete clinical validation data and documentation is available at Variantyx, 1671 Worcester Road, Suite 300 Framingham MA 01701 USA.

## Abbreviations

CAP: College of American Pathologists
CLIA: Clinical Laboratory Improvement Amendments
CMA: Chromosomal Microarray
CNV: Copy Number Variant
FP: False Positive
NGS: Next Generation Sequencing
PAF: Population Allele Frequency
PCR: Polymerase Chain Reaction
SV: Structural Variant
TP: True Positive
WES: Whole Exome Sequencing
WGS: Whole Genome Sequencing
