# Supplementary material for: A clinically validated whole genome pipeline for structural variant detection and analysis
Source: BMC Genomics. 2019 Jul 16;20(Suppl 8):545. doi: 10.1186/s12864-019-5866-z (PMC6631445; doi:10.1186/s12864-019-5866-z)
Supplement: Supplementary file 1 — Table S1 Samples for clinical validation of Variantyx Unity test. Table S2 Variantyx Unity test thresholds. Figure S1 Causative heterozygous deletion of 45 bp detected and reported by Variantyx Unity test. Figure S2 Analytical validation statistics of small sequence changes by Variantyx Unity test basing on combination of 3 different Genome in a Bottle samples. Method S1 Variantyx diagnostic procedure for reporting pathogenic structural variants. (DOCX 152 kb) [file 12864_2019_5866_MOESM1_ESM.docx]

Supplementary materials

Supplementary Table 1. Samples for clinical validation of Variantyx Unity test.

| **Patient ID** | **Type of Variant** | **Patient Phenotype** |
| --- | --- | --- |
| Synthetic panel | 24 variants including 2 SVs | N/A |
| Trisomy1 | Trisomy Chr 21 | N/A |
| Trisomy2 | Trisomy Chr 18 | N/A |
| Trisomy3 | Trisomy Chr 13 | N/A |
| Validation_test_100P | SNV + gross deletion | Neonatal hypotonia, Cardiomegaly, Hepatosplenomegaly, Neurodevelopmental delay, vacuolar myopathy, absence of acid alpha-glucosidase activity in muscle |
| Validation_test_101P | No SV | Vomiting, Diarrhea, Hypoketotic hypoglycemia, Coma, Decreased plasma carnitine |
| Validation_test_102P | No SV | Short stature, Opacification of the corneal stroma, Kyphosis, Grayish enamel, Widely spaced teeth, Platyspondyly |
| Validation_test_103P | No SV | Proximal muscle weakness, Muscular hypotonia, Dyspnea |
| Validation_test_104P | No SV | Decreased beta-galactosidase activity, Normal N-acetylgalactosamine 6-sulfate sulfatase activity |
| Validation_test_105P | No SV | Renal insufficiency, Abnormality of the endocardium, Opacification of the corneal stroma, Sensorineural hearing impairment, Nephrotic syndrome, Abdominal pain |
| Validation_test_106P | Compound het point mutation with large deletion, KRABBE DISEASE, GALC | Decreased nerve conduction velocity, Failure to thrive, Vomiting, Neurodegeneration |
| Validation_test_107P | No SV | Hyperammonemia, Metabolic acidosis, Feeding difficulties in infancy, Muscular hypotonia |
| Validation_test_110P | Alt del in ARSB, MPS VI | parents are related through their grandfather, Limitation of joint mobility, Lumbar hyperlordosis, Depressed nasal bridge, Opacification of the corneal stroma, widely-spaced peg-like teeth, prominent eyes, forehead, and tongue |
| Validation_test_112P | 8k deletion chrX male | MRI of the brain at age 30 was normal; began using a cane at age 28 and was wheel chair bound by age 30, Primary adrenal insufficiency, Spastic paraplegia, loss of vibration sense |
| Validation_test_113P | 8k het deletion GGA compound with splicing poin mutation | Generalized hypotonia, Hepatomegaly, Macroglossia, Cardiomegaly, Increased muscle glycogen content |
| Validation_test_115P | arr Xp11.22(53,581,675-53,692,731)x2,22q11.22q11.23(21,390,449-21,978,719)x1 | Family history suggests X-linked inheritance, Intellectual disability, Macrocephaly |
| Validation_test_116P | arr 2q37.2q37.3(236,218,793-242,654,701)x3,11q25(131,542,057-134,434,130)x1 | Schizophrenia |
| Validation_test_117P | arr[hg19] 5q33.1q35.3(152,281,639-180,686,444)x3,7q36.2q36.3(155,040,999-159,123,167)x1 | Anterior sacral meningocele, Aplasia/Hypoplasia of the sacrum, Presacral teratoma, Holoprosencephaly, Intellectual disability, Mild microcephaly, Short stature |
| Validation_test_118P | Deletion DMD exon 44 | Muscular dystrophy, Calf muscle pseudohypertrophy |
| Validation_test_119P | No SV | Progressive cerebellar ataxia, Dysarthria, Bulbar palsy |
| Validation_test_120P | No SV | Microcephaly, Intellectual disability, severe, Aplasia/Hypoplasia involving the central nervous system |
| Validation_test_121P | No SV | Dementia |
| Validation_test_122P | No SV | Dilated cardiomyopathy |
| Validation_test_123P | No SV | Autism |
| Validation_test_124P | No SV | High nonceruloplasmin-bound serum copper, Cirrhosis, Proteinuria, Kayser-Fleischer ring |
| Validation_test_125P | No SV | Flat face, Protruding tongue, Brachycephaly, Thickened nuchal skin fold, Intellectual disability, Muscular hypotonia, Brushfield spots, Broad palm |
| Validation_test_126P | No SV | Ataxia, Muscular hypotonia, retinitis pigmentosa |
| Validation_test_127P | No SV | Intrauterine growth retardation, Abnormality of cardiovascular system morphology, Micrognathia, clenched fists with overlapping fingers |
| Validation_test_128P | No SV | Global developmental delay |
| Validation_test_129P | No SV | Anencephaly, Spina bifida, Primary adrenal insufficiency |
| Validation_test_201P | Gross deletion | Autism, Speech and developmental delay |
| Validation_test_201R1 | No SV | Non-symptomatic |
| Validation_test_201R2 | No SV | Non-symptomatic |
| Validation_test_204P | Gross deletion | Calf muscle pseudohypertrophy, Elevated serum creatine phosphokinase, Dilated cardiomyopathy, Progressive muscle weakness |
| Validation_test_205P | 53 bp deletion | Hypertension, Impaired platelet aggregation, Thrombocytosis, Splenomegaly |
| Validation_test_206P | No SV | Has done liver biopsy - Hepatocellular cholestasis with balooning degeneration and microvesicular steatosis, Prolonged neonatal jaundice |
| Validation_test_207P | Gross duplication | Age of onset 45, Dementia, Tremor, Glomerulonephritis, Ischemic stroke, Obesity |
| Validation_test_207R1 | No SV | Non-symptomatic |
| Validation_test_207R2 | No SV | Non-symptomatic |
| Validation_test_207R3 | No SV | Non-symptomatic |
| Validation_test_211P | No SV | Cataract, Muscular hypotonia, Myotonia, Skeletal muscle atrophy |
| Validation_test_212P | No SV | Myotonia, Skeletal muscle atrophy |
| Validation_test_214P | No SV | Short stature, Pigmentary retinopathy, Reduced tendon reflexes, Sensorineural hearing impairment, Ptosis |
| Validation_test_215P | CHROMOSOME DELETION | Craniofacial dysmorphia |
| Validation_test_216P | DUPLICATED CHROMOSOME COPY NUMBER VARIATION (CNV) REFERENCE PANEL | Generalized hypotonia, Global developmental delay, dysmorphic facies, incontinentia pigmenti type of skin lesions |
| Validation_test_218P | TRANSLOCATED CHROMOSOME | Abortus fibroblast culture |
| Validation_test_220P | RECOMBINANT CHROMOSOME COPY NUMBER VARIATION (CNV) REFERENCE PANEL 02 | Global developmental delay, Failure to thrive, Congenital heart disease |
| Validation_test_221P | No SV | Dysarthria, Dysmetria, Hyperreflexia, Cone/cone-rod dystrophy, Dysphagia, Babinski sign |
| Validation_test_222P | No SV | Dementia, Behavioral abnormality, Personality changes, Abnormality of the voice, EEG abnormality, Spasticity |
| Validation_test_223P | No SV | Intellectual diability, mild |
| Validation_test_224P | No SV | Intellectual diability, moderate |
| Validation_test_225P | No SV | Premature ovarian failure |
| Validation_test_226P | No SV | One affected son, moderate intellectual disability, Premature ovarian failure |
| Validation_test_227P | No SV | Intellectual diability, mild |
| Validation_test_228P | No SV | Intention tremor |
| Validation_test_229P | No SV | Intention tremor |
| Validation_test_230P | No SV | Intention tremor, MRI findings: white matter lesions involving middle cerebellar peduncles, Gait ataxia |
| Validation_test_231P | No SV | Non-symptomatic |
| Validation_test_232P | No SV | Intention tremor, Orthostatic hypotension, Deficit in phonologic short-term memory, MRI findings: lesions of cerebral white matter |
| Validation_test_241P | No SV | Muscle weakness, Limb girdle and distal inclusion body myopathy |
| Validation_test_241R1 | No SV | Non-symptomatic |
| Validation_test_241R2 | No SV | Non-symptomatic |
| Validation_test_244P | No SV | Distal amyotrophy, Myotonia, Reduced tendon reflexes, difficulty swallowing |
| Validation_test_245P | No SV | Bilateral ptosis, Lower limb muscle weakness, poor ability to swallow cold liquids, difficulty walking on heels, Mother, maternal aunt, two maternal cousins; maternal grandmother (and many of maternal grandmother’s siblings) are also affected. |

Supplementary Table 2. Variantyx Unity test thresholds.

| **Source** | **Name** | **Threshold** |
| --- | --- | --- |
| DNA Extraction and Quality Control | NanoDrop concentration | 30ng/ul |
| DNA Extraction and Quality Control | NanoDrop 260/280 | 1.8 |
| DNA Extraction and Quality Control | NanoDrop 260/230 | 1.8 |
| DNA Extraction and Quality Control | DIN | 7 |
| DNA Extraction and Quality Control | Electrophoresis gel test success | Pass |
| DNA Extraction and Quality Control | Volume sent to sequencer | 60ul |
| DNA Extraction and Quality Control | Concentration sent to sequencer | 30ng/ul |
| DNA Extraction and Quality Control | Qubit | Yes |
| Alignment Quality Metrics | Mapped Reads | 97% |
| Alignment Quality Metrics | Properly Paired Reads Over Total | 93% |
| Alignment Quality Metrics | Properly Paired Reads Over Mapped | 93% |
| Alignment Quality Metrics | QC Failed Reads | 0% |
| Alignment Quality Metrics | Duplicate Reads | 28% |
| Aneuploidy Analysis | Normalized Ploidy | >2.5 or <1.5 autosomes, >1.5 or <0.5 sex chr |
| Aneuploidy Analysis | Predicted Gender | Matches reported gender |
| Aligner | Min Average On-Target Depth | 90% of requested (30X) |
| Aligner | Min On-Target >8x Coverage | 98% |
| Aligner | Min On-Target >0x Coverage | 99% |
| Aligner | Average Template Length | 350 - 850 |
| Aligner | Duplicate Max | 28% |
| Aligner | Global Het VAF Min | 44% |
| Aligner | % Bases with Variants | 0.03% - 0.3% |
| Aligner | Coding Region with Coverage Min | 98% |
| Aligner | Clinically Relevant with Coverage Min | 99% |

Supplemental Figure 1. Causative heterozygous deletion of 45 bp detected and reported by Variantyx Unity test.


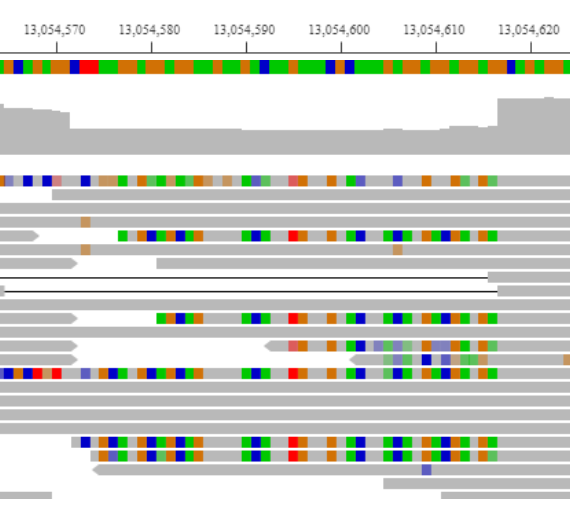


Supplementary Figure 2. Analytical validation statistics of small sequence changes by Variantyx Unity test basing on combination of 3 different Genome in the Bottle samples.


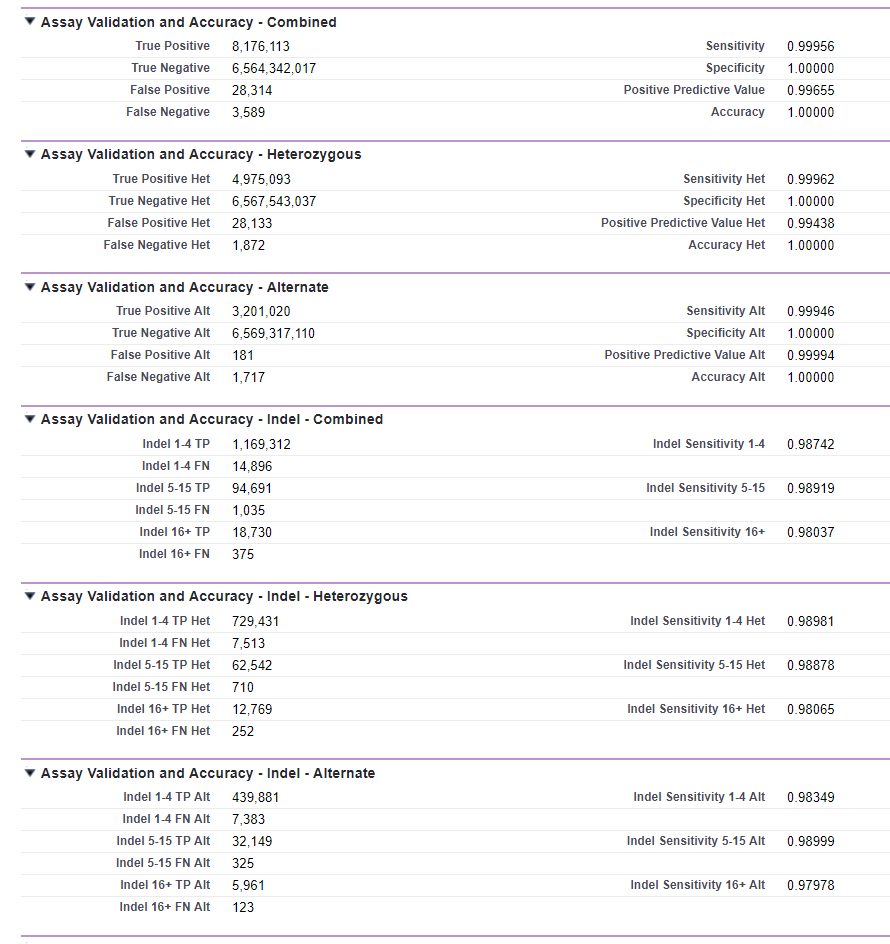


Supplementary Method 1. Variantyx diagnostic procedure for reporting pathogenic structural variants.

- - 1. **Structural variants (Applicable for Whole Genome Sequencing only)**
       1. At the top of the page, click the top left most selector and select “structural variants”
       2. Review and annotate the structural variants:
       3. Review all variants for size; if > than 3 Mb
          1. Note if variant includes any genes or parts of genes
          2. Note if the genes have known function
          3. Map the variant in the Database of Genomic Variants

<http://dgv.tcag.ca/dgv/app/home?ref=GRCh38/hg38>

Make sure you map in Hg38

Copy the CNV location into the search bar in the format: Chr1:123456-125678

Review the genome map to determine if their are any overlapping reported deletions (red) or duplications (blue)

Review in IGV to look for:

Split Reads

Depth Calls

Non-uniquely mapped reads

Poor Quality Reads

- - - 1. Report all variants > 3 Mb even without associated phenotype if
         1. It is not reported in DGV AND
         2. Looks reliable in IGV
      2. Review all variants for size, if < than 3 Mb
         1. Note reported phenotype and gender of the proband
         2. Select structural variants including genes with a

Matching phenotype - gene(s) involved appears to be an explanation for the with reported phenotype (mark as primary)

Overlapping phenotype - gene (s) involved appear to overlap with at least one phentoype or be in the same organ system (mark as secondary)

- - - - 1. Note if allele frequency makes sense with phenotype, age, suspected inheritance and gender of proband

i.e Severe multiple congenital anomalies likely represent a more fully penetrant condition and autosomal dominant and therefore an allele frequency of 1/1000 in healthy populations (DGV)is too high.

I.e. If phenotype is adult onset and more mild or variably penetrant, an allele frequency of 1/1000 in healthy adults in DGV may be plausible due to lack of symptoms at that age or mild symptoms.

I.e. If condition associated with CNV is Autosomal Recessive, check if a second SNV is present; if not, the individual will be a carrier and the CNV not reported

- - - - 1. Note if variant includes any genes or parts of genes
        2. Note if the genes have known function
        3. Map the variant in the Database of Genomic Variants

<http://dgv.tcag.ca/dgv/app/home?ref=GRCh38/hg38>

Make sure you map in Hg38

Copy the CNV location into the search bar in the format: Chr1:12345678-12567890

Review the genome map to determine if their are any overlapping reported deletions (red) or duplications (blue)

- - - - 1. Some structural variants are split into multiple variants so sort by chromosome and check coordinates to identify those in tandem to identify the larger variant
        2. Even if have identified a causative structural variant, continue to review the remaining variants to ensure there are not two or more structural variants.
        3. Classify each selected variant as described in the following two guidelines:

<http://www.nature.com.proxy.hsl.ucdenver.edu/articles/gim92011110.pdf>

<https://www.ncbi.nlm.nih.gov/pmc/articles/PMC5655614/pdf/13353_2017_Article_407.pdf>

Pathogenic

Variant of Uncertain Significance - Likely Pathogenic

Variant of Uncertain Significance

Variant of Uncertain Significance - Benign

Benign

- - - - 1. Report only variants that are in Primary or Secondary AND are classified as Pathogenic, Variant of Uncertain Significance-Likely Pathogenic AND Variant of Uncertain Significance
        2. Do Not Report

Carrier variants

Pathogenic or Likely Pathogenic variants that are UNRELATED to the phenotype other than those that involve the ACMG 59 incidental finding genes < 3 MB

- - - 1. If there are any variants that look relevant but are questionable after reviewing IGV; recommend confirmation with an orthogonal technology on the report
    1. **Report generation**Press the ‘report generation’ button to create a report in docx format. This report will include all of the genetic findings along with any comments and follow up recommendations, disclaimers and variant information. This report is also attached to the bioinformatic analysis.
       1. The Word Document generated should be reviewed for:
          1. Typographical errors
          2. Comparison with TRF data to ensure identifiers are entered accurately
          3. Editing for aesthetics and ease of reading (delete extra spaces, non-grammatically correct imported language etc.)
          4. Inclusion of any pertinent aspects of testing that may not be accounted for i.e. sample of poor quality or other factors that might impact interpretation
